# THE ASSOCIATION BETWEEN COVID-19 ANXIETY AND WELLBEING AMONG UNIVERSITY STUDENTS IN BOSNIA AND HERZEGOVINA

**DOI:** 10.1192/j.eurpsy.2023.1703

**Published:** 2023-07-19

**Authors:** P. Ünal Aydın, S. Trtkovic, O. Aydın, I. S. Ulus, E. Ürek, K. S. Yağmur, B. Ç. Kılavuz

**Affiliations:** Psychology, International University of Sarajevo, Sarajevo, Bosnia and Herzegovina

## Abstract

**Introduction:**

As of September 2022, over 600 million COVID-19 cases have been reported worldwide. Implemented measures and novelty caused by the epidemic caused wellbeing complaints, including depression and anxiety. One particularly inflicted group are students, who were switched to online education. Many universities have decided to start with face-to-face lecturing again, but as the pandemic is still ongoing, the fear of potentially catching COVID and risking one’s wellbeing are still high.

**Objectives:**

The study’s aim is to explore the influence of COVID-19 anxiety on wellbeing among university students.

**Methods:**

A total of 844 university students participated in a 5-minute paper-pen survey, completing self-report scales including a sociodemographic form assessing relevant information regarding COVID-19, The Covid-19 Anxiety Syndrome Scale (C-19ASS) and the Short Warwick-Edinburg Mental Wellbeing Scale (SWEMWBS). Bivariate correlation and multiple linear regression analyses were performed to assess the associations between the variables.

**Results:**

A negative moderate association was found between COVID-19 anxiety and wellbeing, indicating that presence of anxiety related to COVID-19 may predict a lack of wellbeing among university students.

**Conclusions:**

As a negative association between COVID-19 anxiety and wellbeing was found, we can speculate that the existence of anxiety related to COVID-19 may predict student’s wellbeing. Knowing this, different psychological/wellbeing interventions, trainings and techniques, may be utilized to improve the wellbeing of the student population during and after these trying times, to try and minimize the negative effects of the pandemic on the student population.

**Disclosure of Interest:**

None Declared

